# Metabolic dynamics in astrocytes and microglia during post-natal development and their implications for autism spectrum disorders

**DOI:** 10.3389/fncel.2024.1354259

**Published:** 2024-02-14

**Authors:** Iva Cantando, Cristiana Centofanti, Giuseppina D’Alessandro, Cristina Limatola, Paola Bezzi

**Affiliations:** ^1^Department of Fundamental Neurosciences (DNF), University of Lausanne, Lausanne, Switzerland; ^2^Department of Physiology and Pharmacology, University of Rome Sapienza, Rome, Italy; ^3^Istituti di Ricovero e Cura a Carattere Scientifico (IRCCS) Neuromed Via Atinese 18, Pozzilli, Italy

**Keywords:** astrocyte, microglia, mitochondria, brain metabolism, fatty acid oxidation, lipids, lactate, autism spectrum disorders

## Abstract

Autism Spectrum Disorder (ASD) is a complex neurodevelopmental condition characterized by elusive underlying mechanisms. Recent attention has focused on the involvement of astrocytes and microglia in ASD pathology. These glial cells play pivotal roles in maintaining neuronal homeostasis, including the regulation of metabolism. Emerging evidence suggests a potential association between ASD and inborn errors of metabolism. Therefore, gaining a comprehensive understanding of the functions of microglia and astrocytes in ASD is crucial for the development of effective therapeutic interventions. This review aims to provide a summary of the metabolism of astrocytes and microglia during post-natal development and the evidence of disrupted metabolic pathways in ASD, with particular emphasis on those potentially important for the regulation of neuronal post-natal maturation by astrocytes and microglia.

## 1 Introduction

Autism spectrum disorders (ASDs) constitute a complex spectrum of conditions characterized by disruptions in brain development during childhood. The manifestation of neural dysfunction in early life, coupled with the potential for enduring care needs, imposes a substantial burden on society, encompassing social, economic, and medical dimensions. ASDs present a spectrum of conditions stemming from diverse genetic mutations, sharing common phenotypic neuronal alterations that encompass stunted axonal and dendritic arbor growth, deficits in synapse formation, and immature dendritic spines (Ebrahimi-Fakhari and Sahin, 2015). This phenotypic convergence across various ASDs hints at shared molecular mechanisms driving these alterations. While much neurodevelopmental disorders and ASDs research traditionally focused on intrinsic neuronal changes, emerging insights emphasize the pivotal role of alterations in glial cells as well (Petrelli and Bezzi, 2016; Allen and Eroglu, 2017; Cresto et al., 2019; de Oliveira Figueiredo et al., 2022a,b; Ferrucci et al., 2023; Xiong et al., 2023). Indeed, an increasing amount of evidence suggests that disruptions in the intricately balanced interactions among neurons, astrocytes, and microglia can contribute to the onset of ASDs. Astrocytes and microglia play a pivotal role in the regulation of neuronal circuit formation and maturation (Baldwin and Eroglu, 2017; Salter and Stevens, 2017; Stogsdill and Eroglu, 2017; Allen and Lyons, 2018; Thion et al., 2018; Wilton et al., 2019; Thion and Garel, 2020; Cossart and Garel, 2022) and conditions like ASDs have been associated with irregularities in the post-natal maturation of both astrocyte and microglial functions. Maturation delays in glial cells, which encompass mechanisms regulating synapse formation and synaptic homeostasis, are often observed in the context of metabolic dysfunctions occurring since the early phase of post-natal development. While the energy function in the aging brain and related neurodegenerative disorders has been extensively explored (Hou et al., 2019; Bernier et al., 2020; Xiong et al., 2022), there is still limited attention to the metabolic changes occurring in the post-natal period and their implications for ASDs. This is largely due to our limited understanding of the metabolic program unfolding during brain development and the specific nutrient dependencies integral to this process. In this review, we aim to delve into emerging evidence regarding the metabolic insights of glial cells during post-natal development and their potential importance in regulating the maturation of both glial cells and neuronal circuits in the brain.

## 2 Post-natal energy needs of the brain

During post-natal development, the brain undergoes remarkable structural and functional changes, accompanied by significantly heightened energy demands that surpass those of other organs in the body. The newborn human brain, comprising about 13% of body weight, contributes to approximately 60% of the body’s daily energy requirement (Dobbing and Sands, 1973; Bauernfeind et al., 2014; Goyal et al., 2014, 2015; Magistretti and Allaman, 2015; Camandola and Mattson, 2017; Hou et al., 2019; Scheiblich et al., 2020; Bonvento and Bolanos, 2021). Understanding the reasons behind these remarkable energy costs associated with brain function, especially during development, necessitates an exploration of the various components and processes within the brain that incur energy expenditure.

From an evolutionary standpoint, analyzing the glucose and oxygen metabolic rates of the awake adult brain, suggests a linear correlation between the energy consumption and the number of neurons present (Herculano-Houzel, 2011). Recent estimations indicate that neurons alone consume 75–80% of the energy produced in adult brains, with the remaining energy allocated to glial-based processes (Harris et al., 2012; Magistretti and Allaman, 2015; Hyder et al., 2016). The energy demands of neurons arise most from the generation of action potentials and the intricate processes involved in synaptic transmission, including ion fluxes, neurotransmitter release and reuptake, and vesicle cycling (Harris et al., 2012; Magistretti and Allaman, 2015; Hyder et al., 2016).

In the developing brain, it is important to note that the energy requirement during the rapid post-natal growth is due to a swift developmental progression, predominantly driven by the intricate maturation and refinement of existing neurons, as neurogenesis primarily occurs before birth. Indeed, the brain’s adaptability during the post-natal period relies on constant architectural remodeling, involving the addition or elimination of synapses to strengthen or weaken neuronal network activities. This ongoing restructuring necessitates the continuous synthesis and turnover of proteins, lipids, and amino acids, essential for supporting the molecular modifications underlying brain plasticity (Magistretti, 2011; Harris et al., 2012; Bauernfeind et al., 2014; Magistretti and Allaman, 2015; Wilton et al., 2019). Crucially, during rapid post-natal growth, the maturation, and refinement of existing glial cells, including astrocytes and microglia, contribute significantly to energy demands. Astrocytes, originating from neural stem cells, reach their final destinations by migrating along radial glia during early post-natal development (Kriegstein and Alvarez-Buylla, 2009; Ge et al., 2012). Their numbers robustly increase between birth and the third post-natal week (Bandeira et al., 2009; Kriegstein and Alvarez-Buylla, 2009), coinciding with the surge in dendritic and synaptic growth and the maturation of neuronal communication and network properties (Wang and Bordey, 2008; Freeman, 2010; Semple et al., 2013). During post-natal maturation, astrocytes morphogenesis by extending processes to contact blood vessels and synapses and forming the “tripartite synapse” (Araque et al., 1999; Perea et al., 2009; Santello et al., 2012), regulate synaptic connectivity and function and participate in neurotransmitter uptake and release, crucial for fine-tuning synaptic activity (Vesce et al., 1999; Bezzi and Volterra, 2001; Angulo et al., 2008; Araque et al., 2014; Cali et al., 2014; Gómez-Gonzalo et al., 2018; Petrelli et al., 2023). Recent research emphasizes the association between proper astrocyte maturation and the formation, maturation, and pruning of synapses (Stogsdill et al., 2017; Baldwin et al., 2021; Zehnder et al., 2021). For example, astrocytes play a vital role in providing trophic and metabolic factors that support synaptic growth and refinement, including thrombospondins, glypicans and cholesterol (Petrelli and Bezzi, 2016; Baldwin and Eroglu, 2017; Stogsdill and Eroglu, 2017; Allen and Lyons, 2018).

Microglia, the immune cells resident in the central nervous system, begin colonizing the brain from E8.5. They undergo parenchymal proliferation and experience a dieback phase, eventually reaching adult levels of these brain-resident macrophages during the second post-natal week in rodents (Lenz and Nelson, 2018). The principal maturation phase of microglia occurs during post-natal development (Wurm et al., 2021; Zengeler and Lukens, 2021). Initially, they exhibit immature metabolic and molecular features and display an amoeboid morphology. As development progresses, microglia gradually adopt a mature phenotype, characterized by distinct morphological and functional differences compared to their immature form, including a transition to a ramified morphology. This maturation of microglia is crucial for their involvement in a range of neurodevelopmental processes, such as population control, supporting the differentiation and maturation of developing cells, synaptogenesis, neurite outgrowth, axon tract fasciculation, and synaptic pruning (Paolicelli et al., 2011; Thion et al., 2018; Prinz et al., 2019; Thion and Garel, 2020; Cossart and Garel, 2022; Guedes et al., 2023).

## 3 Post-natal brain metabolism and the metabolic flexibility of astrocytes and microglial cells

To satisfy its substantial energy requirements, the mature brain primarily depends on glucose as its main energy source (Dienel, 2019). Astrocytes play a significant role in glucose uptake, a function attributed to their abundant expression of glucose transporters, gap junction channels, and glucose-metabolizing enzymes within their perivascular domains (Rouach et al., 2008; Allaman et al., 2011; Escartin and Rouach, 2013; Magistretti and Allaman, 2015). It is now understood that astrocytes, rather than neurons, are the primary generators of energy through the glycolytic pathway (Bélanger et al., 2011; Magistretti and Allaman, 2015). They express specific glycolytic enzymes, enabling them to obtain 80% of their glucose supply through glycolysis. In contrast, neurons, with inhibited enzymes such as 6-phosphofructo-2-kinase/fructose-2,6-biphosphatase 3 (PFKFB3) and pyruvate dehydrogenase kinase 4 (PDK4), are more reliant on phosphorylation (Magistretti and Allaman, 2018). Notably, astrocytes predominantly express lactate dehydrogenase 5 (LDH5), which facilitates the conversion of pyruvate to lactate, while neurons exclusively express LDH1, favoring the reverse reaction (Bittar et al., 1996). Additionally, the higher NADH to NAD + ratio in astrocytes further promotes the reduction of pyruvate to lactate (Mongeon et al., 2016). Astrocytes respond to neuronal activity by taking up glutamate, which can be recycled through the glutamate-glutamine cycle (Bak et al., 2006; McKenna, 2007). Recent studies, such as that by Cheung et al. (2022), have highlighted the indispensability of this cycle for synaptic transmission and memory. The uptake of glutamate triggers aerobic glycolysis, leading to the secretion of lactate. In accordance with the astrocyte-neuron lactate shuttle (ANLS) model (Pellerin and Magistretti, 1994; Bélanger et al., 2011; Magistretti and Allaman, 2018), neurons utilize these extracellular monocarboxylates, channeling them into the mitochondrial respiratory chain and oxidative phosphorylation (OXPHOS) processes to ultimately produce energy.

Mature microglial cells, like astrocytes, are capable of glucose uptake. However, unlike astrocytes, microglia primarily depend on oxidative glycolysis. This understanding stems from various cell type-specific transcriptomic studies, which have shown that microglia express transporters and enzymes necessary for glucose metabolism (Zhang et al., 2014; Bennett et al., 2016; Zeisel et al., 2018). Among the multiple glucose transporter (GLUT) isoforms transcribed in microglia, GLUT3 is notable for being highly expressed, similar to its expression in neurons. The reliance of microglia on oxidative glycolysis has been suggested by comprehensive functional studies, particularly those examining the metabolic pathways that support their continuous and dynamic motility during the resting surveillance phase. Initial studies involving microglial cell lines or primary microglia in meticulously controlled cell cultures have provided valuable insights into the specific nutritional needs of microglia. However, it’s important to recognize that cultured microglia display distinct transcriptional signatures and phenotypes, markedly different from those in their natural *in vivo* environment (Kettenmann et al., 2011; Butovsky et al., 2014; Bohlen et al., 2017). The utilization of oxidative pathways by adult microglial cells has been recently corroborated by transcriptomic studies. These studies indicate that mouse microglia transcribe most of the genes necessary for OXPHOS, thereby confirming their primary reliance on oxidative metabolism of glucose (Zhang et al., 2014; Bennett et al., 2016; Orihuela et al., 2016; Ghosh et al., 2018).

**FIGURE 1:**
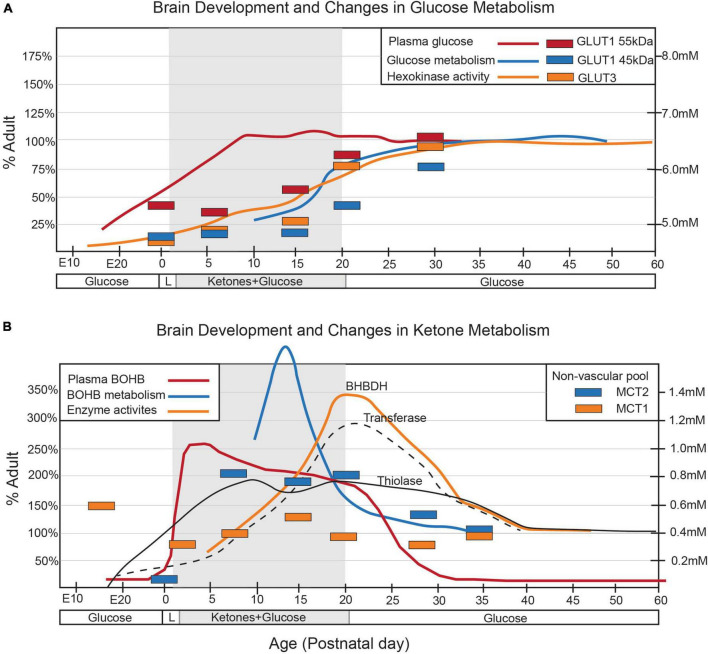
Alterations in arterial concentrations, cerebral enzyme activities, and cerebral transporters for **(A)** glucose and **(B)** ketone metabolism throughout postnatal development are depicted in the graphs. The shaded area in each graph corresponds to the suckling period. Changes in arterial concentrations (solid lines) are represented by the mmol/L values on the y-axis (right). Enzymatic activities (broken lines) and changes in transporter density (bars) are presented as a percentage of the adult levels. Figure modified from Vannucci et al., 1994 and Vannucci and Simpson, 2003.

The metabolism of the developing brain in suckling mammals significantly differs from that of mature individuals (Figure 1). During lactation, and with the post-natal consumption of high-fat milk, ketone bodies (KBs), derived from fatty acids and produced by the liver, provide much of the energy required by the neonatal brain (Cremer, 1982; Dombrowski et al., 1989; Vannucci and Vannucci, 2000; Nehlig, 2004; Oyarzábal et al., 2021). Therefore, the key factors in energy metabolism during neurodevelopment include glucose, lactate, and KBs (Oyarzábal et al., 2021).

**FIGURE 2:**
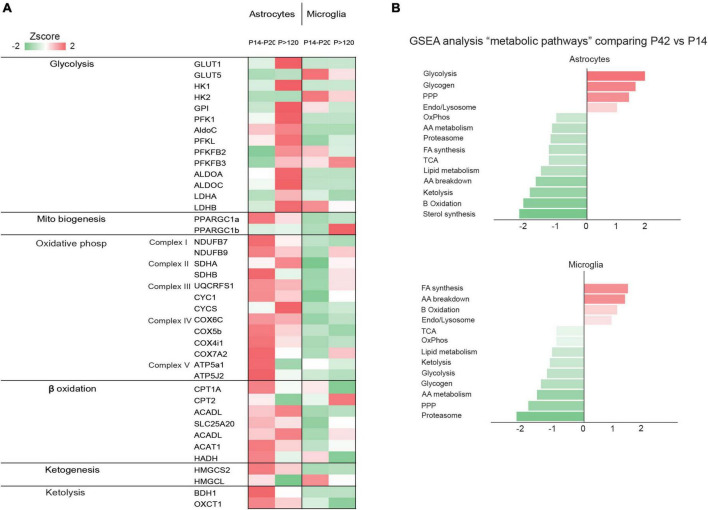
**(A)** The heatmap shows the gene expression levels of glycolytic enzymes in astrocytes, neurons, microglia, endothelial cells, and oligodendrocytes. Data obtained from published data by Zhang et al. (2014, 2016); Bennett et al. (2016); Clarke et al. (2018); Düking et al. (2022); **(B)** The graph shows normalized enrichment score of the “metabolic pathways” gene sets by gene set enrichment analysis, comparing z-scores of astrocyte and microglia proteomes from P42 vs. P14. The figure is a modified version of Figure 1D in Düking et al. (2022).

Although glucose is the primary energy source in the adult brain, its role in the neonatal brain is relatively subdued despite similar bloodstream levels to adults (Vannucci and Vannucci, 2000; Brekke et al., 2015). This is partly due to the limited number of GLUTs present during this developmental stage, which restricts glucose use in early post-natal development (Vannucci et al., 1994; Vannucci and Simpson, 2003). GLUT1, responsible for transporting glucose across the blood-brain barrier (BBB), is mainly expressed by endothelial cells and astrocytes, while GLUT3 is primarily expressed by neurons (Vannucci et al., 1994; Zhang et al., 2016; Caldwell et al., 2022). After weaning, when mammals switch to carbohydrate-based nutrition, the expression of both GLUT1 and GLUT3 increases significantly (Figure 2), coinciding with glucose replacing lactate and KBs as the main energy substrate (Zhang et al., 2014, 2016; Caldwell et al., 2022; Düking et al., 2022). Little is known about the glucose metabolism of microglial cells during post-natal development, but recent meta-analyses of transcriptomic and proteomic studies suggest that developing mouse microglia transcribe most of the genes necessary for glycolysis, implying a predominant reliance on glycolytic glucose metabolism (Zhang et al., 2014; Bennett et al., 2016; Ghosh et al., 2018). The post-natal expression of various GLUT isoforms, including GLUT3 and GLUT5, suggests an ability to utilize different sugars. Notably, in the central nervous system, microglia appear to be the only cells expressing the GLUT5 hexose transporter, predominantly specific for fructose. However, given the limited availability of fructose in the brain under physiological conditions, the functional role of GLUT5 remains unclear (Payne et al., 1997; Douard and Ferraris, 2008; Hwang et al., 2017). Under inflammatory conditions, GLUT1 expression is upregulated in microglia (Wang et al., 2019), increasing glucose uptake and promoting glycolysis, which underscores how metabolic changes contribute to modulating microglial homeostasis (Lauro and Limatola, 2020). The primary rate-limiting enzymes in glucose metabolism are hexokinases (HKs), which catalyze the phosphorylation of glucose to glucose-6-phosphate (Wilson, 2003, 2006). Of the four main HK isozymes (HK1-4), each has distinct biochemical features and catalytic activities (Wilson, 2003, 2006). HK1 and HK2, historically recognized as primarily expressed in the brain and muscle/adipose tissues, respectively, (Wilson, 2006), are associated with the outer mitochondrial membrane, facilitating access to mitochondrial ATP to promote glycolysis (Wilson, 2006; Pastorino and Hoek, 2008). Recent research has identified selective HK2 expression in microglia, predominantly in neurons and astrocytes (Hu et al., 2022). A transcriptome meta-analysis revealed that microglial HK2 levels peak in the early phase of post-natal development (P7-P14) (Zhang et al., 2016; Clarke et al., 2018; Düking et al., 2022; Figure 2). A study in rodents demonstrated that genetic ablation of HK2 leads to decreased microglial glycolytic flux and energy production, inhibiting microglial repopulation, attenuating surveillance, and impairing migration triggered by damage (Hu et al., 2022). These findings suggest a crucial role for microglial glycolysis in the post-natal brain.

**FIGURE 3:**
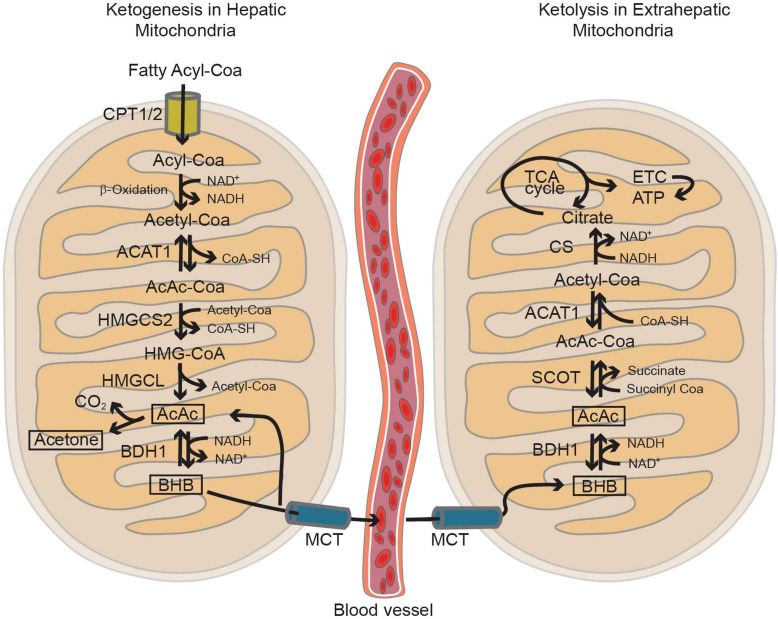
The mitochondria play a key role in both ketogenesis and ketolysis pathways. Ketogenesis primarily takes place in hepatic mitochondria, utilizing acetyl-CoA generated through the β-oxidation of fatty acyl-CoA. Subsequently, extrahepatic tissues absorb these ketone bodies, such as β-hydroxybutyrate (BHB) and acetoacetate (AcAc), via the monocarboxylic acid transporter (MCT). BHB can fuel the brain as alternative energy substrates under non-physiological conditions such as starvation, insulin-resistance and during post-natal development. In the mitochondria of the brain cells, ketolysis occurs, converting BHB and AcAc back into Acetyl-CoA. This process generates ATP through the tricarboxylic acid (TCA) cycle and the electron transport chain (ETC). Figure modified from Hwang et al. (2022).

It is believed that the perinatal brain is greatly reliant on the metabolism of lactate and KBs, but this reliance seems to diminish gradually as the use of glucose increases (Vannucci and Vannucci, 2000) and the precise rate, timing, and triggers of the shift to glucose metabolism has been extensively investigated (Medina and Tabernero, 2005). Rodent brain actively uses lactate during the fetal stage, early neonatal period, and suckling phase (Medina and Tabernero, 2005) but, throughout the perinatal period, various other nutrients such as glucose, amino acids, and fatty acids are transplacentally transferred from the mother and can easily enter the brain through the still incompletely formed BBB (Burd et al., 1975). The role of lactate seems to be particularly crucial in the post-natal period, when the brain continues its developmental trajectory by forming and refining the synapses (Medina and Tabernero, 2005). The post-natal synthesis of KBs (i.e., ketogenesis) primarily occurs in hepatocytes (Puchalska and Crawford, 2017), but the use of KBs as metabolic substrates (i.e., ketolysis) predominantly involves peripheral tissues including the brain (Puchalska and Crawford, 2017; Figure 3). The KBs in developing brain cells enter the mitochondria for ketolysis and supply aceto- and acetoacetyl-CoA for the tricarboxylic acid (TCA) cycle, ATP production, and the synthesis of lipids, fatty acids, and cholesterol (Yeh and Sheehan, 1985). Brain entry and the intercellular transfer of lactate and KBs is a highly regulated process that is mediated by monocarboxylate transporters (MCTs), which are particularly abundant in the BBB and endothelial cells (Leino et al., 1999; Rafiki et al., 2003; Kishimoto et al., 2016). MCT1, MCT2, and MCT4 are the most abundant in the developing brain and can all shuttle lactate and KBs, albeit with different affinities (Halestrap, 2013). The different MCTs are differently expressed in producing and receiving cells: lactate-receiving neurons express the high-affinity MCT2, whereas quasi-producing astrocytes express the lower-affinity MCT1 and MCT4 (Pellerin et al., 1998a,b; Rafiki et al., 2003; Kasischke, 2011; Kishimoto et al., 2016; Zhang et al., 2016; Roosterman and Cottrell, 2020; Caldwell et al., 2022; Düking et al., 2022). Furthermore, although not extensively studied, it has been recently reported that pathologically affected microglia also express MCTs (Moreira et al., 2009; Ding et al., 2013; Nijland et al., 2014). It is also interesting to note that some MCTs are highly expressed during post-natal development: for example, MCT1 levels peak between P4 and P14 (Hanamsagar et al., 2017) thus further suggesting that such cells may depend on lactate and KBs metabolism during their post-natal maturation. Transcriptomic analysis has also revealed that MCT4 levels are higher in microglia than in other brain cell types (Zhang et al., 2014; Bennett et al., 2016; Clarke et al., 2018; Düking et al., 2022). In order to determine whether astrocytes and microglia preferentially use MCTs for lactate or KBs, it is necessary to consider the expression of enzymes such as lactate dehydrogenase (LDH), which facilitates the reversible conversion of pyruvate and NADH to lactate and NAD + in a dynamic equilibrium (Rogatzki et al., 2015). LDH exists in the form of homotetramers or heterotetramers consisting of two distinct subunits: LDHA and LDHB (the former being primarily responsible for catabolizing pyruvate into lactate and the latter for catabolizing lactate into pyruvate) (Rogatzki et al., 2015; Urbańska and Orzechowski, 2019). Consequently, in conjunction with other factors, the varied composition of LDH tetramers can influence the direction of the reaction toward lactate catabolism or production. Intriguingly, transcriptomic analysis has shown that mRNA levels of LDHB are particularly high in microglial cells and reach their zenith in the early post-natal period (P14), a phase marked by substantial microglia-dependent synaptic remodelling, whereas LDHA expression is minimal (Bennett et al., 2016; Figure 2), thus indicating that microglia predominantly engage in lactate oxidation rather than lactate production during this developmental window. It has been discovered that microglia efficiently uptake lactate, leading to lysosomal acidification (Monsorno et al., 2023). Among key lactate transporters in the brain, microglial MCT4 is dynamically regulated in response to exogenous lactate. MCT4 is crucial for lactate-dependent lysosomal modulation in microglia, and cells lacking MCT4 show deficits in cargo uptake and degradation. In early post-natal development, conditional knockout mice (cKO) with selective microglial MCT4 depletion exhibit impaired synaptic pruning and elevated synaptic marker levels in the CA1 hippocampal region. Functionally, hippocampal pyramidal neurons in cKO mice demonstrate greater excitatory drive, evidenced by larger excitatory post-synaptic currents and an increased excitatory/inhibitory (AMPA/GABA) ratio. Furthermore, juvenile mice lacking microglial MCT4 are prone to kainic acid-induced seizures, suggesting circuit hyperexcitability. In adulthood, MCT4 cKO mice display an anxiety-like psychiatric phenotype. These findings collectively indicate that disrupting microglial MCT4 disrupts the refinement of microglia-mediated synapses, leading to defective brain development and altered adult behavior (Monsorno et al., 2023).

In contrast to microglial cells, astrocytes express high levels of LDHB and other glycolytic enzymes in the later stages of post-natal development when their maturation is nearly complete (Zhang et al., 2016; Caldwell et al., 2022; Düking et al., 2022; Figure 2). Increasing evidence suggests that the metabolism of developing astrocytes is more aligned with mitochondrial function than glycolysis (Zhang et al., 2016; Zehnder et al., 2021; Caldwell et al., 2022; Düking et al., 2022). Although the importance of mitochondrial activity in developing astrocytes is not fully understood, recent studies have shown that mitochondria in mature astrocytes are vital for maintaining homeostatic functions of associated synapses (Motori et al., 2013; Ignatenko et al., 2018; Göbel et al., 2020). Notably, there is a significant increase in mitochondrial occupancy throughout post-natal astrocyte development (Zehnder et al., 2021). Similar to cells with high oxidative capacity, developing astrocytes are enriched in PGC-1α and PGC-1β (Puigserver and Spiegelman, 2003). Consistent with the role of PGC-1α in regulating mitochondrial biogenesis and respiratory function (Wu et al., 1999), its removal from astrocytes reduces mitochondrial content and decreases the expression of genes essential for the electron transport chain (ETC) and OXPHOS, affecting mitochondrial respiration (Zehnder et al., 2021). These findings support a transient need for mitochondrial biogenesis in developing astrocytes, especially during the period marking the end of proliferation and the start of functional and morphological maturation. Complementing this, transcriptome and proteome analyses reveal high levels of mRNA and proteins involved in mitochondrial ketolysis (SCOT1, SCOT2, ACAT1) and ketogenesis (CPT1A, HMGCS2, BDH1) during the suckling period, which decrease after weaning (Auestad et al., 1991; Blázquez et al., 1998; Guzmán and Blázquez, 2001; Zhang et al., 2016; Eraso-Pichot et al., 2018; Fecher et al., 2019; Caldwell et al., 2022; Düking et al., 2022; Silva et al., 2022; Figure 2). This metabolic shift stems from astrocytes’ ability to store and oxidize fatty acids (FAs), suggesting a system for local production and delivery of KBs within neighboring cells, including neurons. The contribution of astrocyte-derived KBs to neuronal oxidation, particularly in supporting memory formation, has been demonstrated *in vivo* in *Drosophila melanogaster* undergoing starvation (Silva et al., 2022). This highlights the adaptive mechanisms used by neurons under challenging metabolic conditions and underscores the significance of KBs as an essential energy substrate, even in the absence of glucoses (Chowdhury et al., 2014; Silva et al., 2022). Further, studies have revealed that developing astrocytes are not only capable of mitochondrial FAs oxidation but also play a role in detoxifying excess neuron-derived FAs (Civenni et al., 1999; Ioannou et al., 2019; Qi et al., 2021). Neuronal cells, particularly those that are highly active, generate excessive FAs but struggle to utilize them for oxidative ATP synthesis, leading to the accumulation of toxic FAs. To mitigate neuronal damage, these excess FAs are stored in astrocytes’ intracellular lipid droplets (LDs) as triglycerides. Apolipoproteins assist in transporting excess FAs into astrocytes, which, with their abundant LDs, are less vulnerable to the harmful activity of reactive oxygen species (ROS) compared to neurons. LDs in astrocytes serve as energy storage depots, transferring FAs to mitochondria during nutrient depletion and acting as an alternative energy source when consumed. Therefore, the processes of FAs storage and oxidation depend on a close metabolic interconnection between neurons and astrocytes (Ebert et al., 2003; Ioannou et al., 2019). It appears that genes related to FAs synthesis, β-oxidation, and lipid metabolism are more highly expressed in astrocytes during the suckling period than during weaning (Zhang et al., 2016; Clarke et al., 2018; Caldwell et al., 2022; Düking et al., 2022; Figure 2), indicating that astrocytic lipid metabolism may be critical during this neonatal stage. Given the similarity of the diet of suckling mammals to a ketogenic diet, characterized by high FAs and low carbohydrate levels (García-Rodríguez and Giménez-Cassina, 2021), FAs may be the primary energy source for astrocytes. A recent study demonstrated that astrocyte-specific deletion of carnitine-palmitoyl transferase-1A (CPT1A), a key enzyme in mitochondrial FAs oxidation, leads to cognitive impairment in mice (Morant-Ferrando et al., 2023). The underlying mechanism involves a shift in astrocytic pyruvate metabolism that ultimately promotes a reduction in ROS, which have been shown to provide a crucial signal in astrocytes capable of modulating brain metabolism and sustaining murine behavioral performance (Vicente-Gutierrez et al., 2019).

Unlike fatty acids, which maintain equilibrium with the rest of the body, almost all brain cholesterol is synthesized by astrocytes (Pfrieger and Ungerer, 2011). This is because cholesterol-carrying lipoproteins, except for some very dense high-density lipoproteins (HDLs), cannot readily cross the BBB (Balazs et al., 2004). Astrocytes play a crucial role in brain cholesterol metabolism. For instance, when neuronal-like retinal ganglion cells are cultured in glia-conditioned media, an increase in synapse formation is observed, with cholesterol identified as a key mediator of this effect (Mauch et al., 2001). This suggests that astrocytes synthesize and transport cholesterol to neurons via lipoprotein particles. The importance of this mechanism is further highlighted by the fact that deleting LRP1, the primary receptor for Apolipoprotein E (ApoE) on neurons, results in impaired dendritic spine development and neurodegeneration with aging (Liu et al., 2010). Cholesterol levels are tightly regulated by sterol regulatory element-binding protein 2 (SREBP2), the major transcription factor for genes involved in cholesterol synthesis (Brown and Goldstein, 2009). RNA profiling of mouse and human brain astrocytes, both through bulk sequencing (Zhang et al., 2014, 2016; Chai et al., 2017) and single-cell sequencing (Batiuk et al., 2020; Endo et al., 2022), has revealed high expression levels of SREBP2 and 12 other genes involved in cholesterol biosynthesis in developing astrocytes (Pfrieger and Ungerer, 2011; Valenza et al., 2023). This suggests a significant role for astrocytes in cholesterol production and metabolism. Studies involving astrocyte-specific inactivation of SREBP-mediated lipid biogenesis in mice demonstrate that reduced SREBP activity in astrocytes hinders presynaptic terminal development and impairs hippocampal function, likely due to decreased levels of the presynaptic protein SNAP-25 and fewer synaptic vesicles (van Deijk et al., 2017). Furthermore, inactivating SREBP2, the key regulator of cholesterol synthesis genes in astrocytes, leads to reduced brain size in mice, particularly in astrocyte-rich regions (Ferris et al., 2017). Excess cholesterol is converted into cholesterol esters through the biosynthetic activity of two distinct genes: acyl-CoA:cholesterol acyltransferase 1 (Acat1) and 2 (Acat2), also known as sterol O-acyltransferase 1 and 2 (Soat 1,2). Transcriptome and proteomic analyses indicate that both ACAT1 and ACAT2 enzymes, encoded by these genes, are highly expressed in astrocytes during post-natal development (Zhang et al., 2016; Caldwell et al., 2022; Düking et al., 2022; Figure 2). These enzymes, located in the endoplasmic reticulum (ER) and enriched at the mitochondria-associated ER membrane, use long-chain fatty acyl-CoAs and sterols with 3-beta-OH, including cholesterol and various oxysterols, as substrates (Liu et al., 2005). They are allosterically activated by cholesterol or oxysterols. Notably, activation of ACAT1/SOAT1 in astrocytes occurs under conditions with excessive cholesterol content or a lack of ApoE, leading to augmented lipid storage and inflammatory processes (Karten et al., 2006).

**FIGURE 4:**
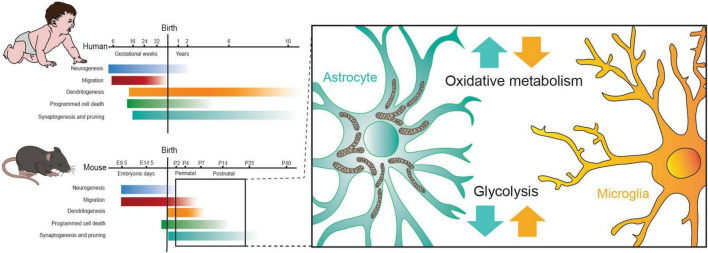
The alteration in metabolism of astrocytes and microglia observed during the perinatal critical period aligns with the onset of dendritogenesis, synaptogenesis and pruning in mice. Figure modified from Oury and Pierani (2023).

In conclusion, the metabolic landscape of the developing brain is marked by dynamic shifts in its preference for energy substrates, with astrocytes and microglia playing pivotal yet distinct roles at various developmental stages. While glucose serves as the primary energy source in the mature brain, the neonatal brain initially relies on lactate and ketone bodies (KBs), gradually transitioning to a greater dependence on glucose. Astrocytes, during post-natal development, show a predilection for mitochondrial metabolism, including FAs oxidation and ketolysis. In contrast, microglial cells depend on glycolysis, facilitated by the expression of a variety of glucose transporters and enzymes (Figure 4). Understanding the intricate interactions between astrocytes and microglia in meeting the energy requirements of developing brain neurons is essential for advancing our comprehension of the mechanisms that drive the functional and structural maturation of neuronal circuits.

## 4 Dysregulation of metabolic pathways in ASDs: exploring implications for glial cell metabolism

Although the cause of ASDs is identified in only <10% of cases (Lord et al., 2020), it’s believed that their etiology involves a complex interplay of multigenic interactions and environmental, infectious, metabolic, and nutritional factors (Niemi et al., 2018; Tãrlungeanu and Novarino, 2018; Lord et al., 2020). However, the underlying biological mechanisms remain largely unclear. A growing body of evidence suggests that dysregulated glial cells can contribute to many of the neurological symptoms observed in various neurodevelopmental disorders (Petrelli et al., 2016, 2023; Neniskyte and Gross, 2017; Petrelli and Bezzi, 2018; Cresto et al., 2019; de Oliveira Figueiredo et al., 2022a,b; Ferrucci et al., 2023; Matuleviciute et al., 2023; Xiong et al., 2023). For instance, while genes implicated in ASDs were initially thought to be exclusively expressed in neurons, recent findings show their expression in astrocytes and microglial cells as well, suggesting a potential role for these cells in the development of these disorders (McGann et al., 2012; Petrelli et al., 2016; Petrelli and Bezzi, 2018; de Oliveira Figueiredo et al., 2022b; Ferrucci et al., 2023). Moreover, as discussed earlier, energy metabolism is crucial in normal brain development, and it has been demonstrated that metabolic irregularities contribute significantly to various neurodevelopmental and psychiatric disorders (Cheng et al., 2017; Graham et al., 2020; Oyarzábal et al., 2021; Žigman et al., 2021; Kotchetkov et al., 2023). The metabolic pathways of astrocytes and microglial cells undergo significant adaptations during post-natal development due to their reliance on a milk-based, high-fat diet. These cells over-express many key pathways responsible for maintaining energy in developing post-natal brain cells, suggesting a pivotal role in ensuring the energy required for the development of brain circuits. The following section will delve into brain energy dysfunctions associated with ASDs, with a particular focus on those that, even if not directly documented, could affect astrocytes and microglial cells, and consequently, the maturation of neuronal circuits.

ASDs are heterogeneous, highly heritable neurodevelopmental conditions, characterized by deficits in sociability and communication, as well as the presence of restrictive and repetitive behavioral patterns (Lord et al., 2020). The diversity of molecular pathways associated with ASDs reflects the complexity of their etiology (Lord et al., 2020), and deciphering their genetic-environmental interactions provides valuable insights into their biological underpinnings. Metabolic disruption and mitochondrial dysfunctions are notably more prevalent in ASD patients compared to the general population, suggesting a potential involvement of mitochondrial metabolism in ASD development, although its specific role remains elusive (Rossignol and Frye, 2012; Hollis et al., 2017).

The hypothesis that mitochondrial dysfunctions may contribute to ASDs has been evolving since 1985 when lactic acidosis was observed in 5% of autistic patients (Coleman and Blass, 1985). Subsequent studies have indicated that altered ETC activity in ASD patients could lead to decreased ATP production and increased oxidative stress, variably impacting different brain regions or cell types (Hollis et al., 2017). This aligns with findings that nearly one-third of autistic children exhibit high plasma lactate levels and/or a high lactate-to-pyruvate ratio (Haas, 2010; Rossignol and Frye, 2012; Oh et al., 2020), though it’s yet to be determined whether these elevated lactate levels are a cause or a result of autism. The heightened brain lactate levels in ASDs may arise from increased glycolysis, diminished TCA cycle activity, and restricted OXPHOS. Recent research further refines this perspective by highlighting the role of astrocytes in mitochondrial dynamics during development. Studies have found increased mitochondrial biogenesis and occupancy in developing astrocytes compared to their mature counterparts (Zehnder et al., 2021), attributed to higher levels of PGC-1α, a key regulator of mitochondrial biogenesis and function, particularly during the second week of post-natal development (Zhang et al., 2014, 2016; Clarke et al., 2018; Zehnder et al., 2021; Düking et al., 2022). The removal of PGC-1α leads to reduced mitochondrial content, elevated extracellular lactate levels, and a decrease in the number of excitatory synapses, resembling the conditions observed in many neurodevelopmental disorders and ASDs. This suggests a critical need for robust mitochondrial biogenesis in developing astrocytes, particularly for synaptic formation. While the exact mechanism by which astrocytic mitochondria regulate excitatory synapse formation remains to be elucidated, the data indicating high mRNA levels of genes controlling mitochondrial biogenesis and functions (including oxidative phosphorylation, ketolysis, and ketogenesis/FA oxidation) in developing astrocytes, suggest a crucial role in supporting neuronal energy metabolism. Any impairment in astrocytic mitochondrial functions, as indicated by altered PGC-1α activity, could contribute to the mitochondrial dysfunctions observed in neurodevelopmental disorders and ASDs. Therefore, a deeper understanding of the role of PGC-1α in astrocyte mitochondrial function could provide significant insights into the mitochondrial aspects of ASD pathophysiology, particularly in terms of the metabolic support that astrocytes offer to neurons. The understanding of lactate’s role in ASDs is further expanded by recent insights into the function of microglial MCT4. Transcriptomic analysis has revealed that during early post-natal development, a critical period for microglia-dependent synaptic remodeling, microglial cells express high levels of LDHB, suggesting a predilection for lactate oxidation rather than production (Bennett et al., 2016; Monsorno et al., 2023). Microglia efficiently uptake lactate, which is crucial for lactate-dependent lysosomal modulation, as evidenced by MCT4’s role in these cells. MCT4’s dynamic regulation in response to exogenous lactate and its significance in synaptic pruning and neuronal excitability further underscore its importance. For instance, microglial MCT4 depletion in conditional knockout mice results in impaired synaptic pruning, elevated synaptic marker levels in the CA1 hippocampal region leading to circuit hyperexcitability and anxiety-like behavior in adulthood (Monsorno et al., 2023). These findings collectively suggest the dysregulation of lactate oxidation in microglia could disrupt synaptic development and neuronal function, contributing to the neurodevelopmental and behavioral manifestations associated with ASDs. Therefore, understanding the interplay between lactate metabolism and microglial function could provide valuable insights into the metabolic underpinnings of ASDs and potentially reveal novel therapeutic targets.

Our understanding of the mitochondria-ASD relationship has expanded in the last years through numerous studies (Rossignol and Frye, 2012; Hollis et al., 2017; Frye, 2020) identifying several genes encoding mitochondrial proteins as ASD risk genes as well as impaired mitochondrial function in the brains of ASD patients. These studies, including animal models, suggest that systemic mitochondrial mutations might cause tissue-specific brain defects and regional neurophysiological alterations, leading to autistic endophenotypes (Kanellopoulos et al., 2020; Yardeni et al., 2021). A comprehensive meta-analysis has revealed that the prevalence of abnormal mitochondrial function biomarkers often exceeds the overall 5% prevalence of classical mitochondrial disease (Rossignol and Frye, 2012), with significant findings such as high levels of lactate in 31% of cases, pyruvate (14%), alanine (8%), and others. Moreover, a striking 80% of children with ASD exhibit low carnitine levels (Mostafa et al., 2005; Frye, 2020). Carnitine, crucial for mitochondrial FA oxidation and energy production, is primarily processed by astrocytes during post-natal brain development. Evidence points to abnormalities in mitochondrial FA oxidation in a subset of children with ASDs. This includes extensive documentation of free L-carnitine depletion, essential for transporting FAs into the mitochondria (Mostafa et al., 2005), a reduction in mitochondrial FA β-oxidation (Mitochondrial Medicine Society’s Committee on Diagnosis, 2008), elevated levels of long- and very-long-chain FAs in serum, and increased levels of acyl-carnitine, a biomarker of deficient mitochondrial FA oxidation (Frye, 2012; Frye et al., 2013). A deficiency in L-carnitine may be linked to reduced expression of the TMLHE gene, encoding a mitochondrial protein crucial for carnitine biosynthesis and expressed by astrocytes during post-natal development (Zhang et al., 2014). The involvement of TMLHE in the onset of ASDs is supported by findings such as exon 2 deletion in males with ASD (Ziats et al., 2015) and the association of TMLHE deficiency with high ε-N-trimethyl lysine levels, a recognized risk factor for ASDs (Celestino-Soper et al., 2012). While its specific role in immature astrocytes has not been directly investigated, one can infer its potential impact based on the gene’s known functions and the critical role of astrocytes in brain development. In immature astrocytes, TMLHE may be crucial for maintaining adequate levels of L-carnitine, which is essential for the transport of long-chain FAs into the mitochondria for β-oxidation. Given the high energy demands of the developing brain, particularly for processes like synaptic formation and myelination, any impairment in TMLHE function could lead to insufficient carnitine levels, thereby hindering mitochondrial FAs metabolism in astrocytes. This could result in energy deficits and the accumulation of unmetabolized FAs, potentially contributing to the disrupted neural development observed in ASDs. Furthermore, the role of TMLHE in regulating ε-N-trimethyl lysine levels, a risk factor for ASDs, suggests that abnormalities in TMLHE expression or function could disrupt metabolic homeostasis in astrocytes. This disruption might impact astrocyte-neuron interactions and the overall neurodevelopmental environment, possibly exacerbating or contributing to the pathophysiology of ASDs. Therefore, exploring the role of the TMLHE gene in the metabolism of immature astrocytes could provide valuable insights into its contributions to the complex etiology of ASDs, particularly in the context of mitochondrial function and metabolic regulation.

The potential involvement of glial cells in the development of ASD-related deficits extends to lipid metabolism. Lipids play a critical role in various aspects of neuronal development, including migration, differentiation, morphogenesis, myelination, memory formation, and synaptic plasticity, all of which are crucial for proper neurodevelopment (Salvati et al., 2000; Wang and Eckel, 2014). These processes are especially pertinent to ASDs, as they significantly influence neurodevelopmental trajectories (de la Torre-Ubieta et al., 2016; Usui et al., 2017; Heavner and Smith, 2020). Numerous studies have highlighted the importance of lipid metabolism in the pathophysiology of ASDs. For example, Smith–Lemli–Opitz syndrome (SLOS), an inborn error in cholesterol synthesis resulting from mutations in the 7-dehydrocholesterol reductase (DHCR7) gene, manifests as developmental delays, abnormal neural development, and atypical peripheral lipid metabolism (Bukelis et al., 2007; Porter, 2008). Notably, the DHCR7 gene, linked to SLOS, is highly expressed in astrocytes during early post-natal development (Zhang et al., 2014, 2016), underscoring the potential impact of this gene on astrocyte function. Recent research has delved into the effects of DHCR7 mutations on astrocytes (Freel, 2022). Studies involving DHCR7 mutant mice have revealed that astrocytes in these animals exhibit hallmark signs of reactivity, such as increased expression of glial fibrillary acidic protein (GFAP) and cellular hypertrophy. Transcript analysis has shown extensive immune activation in these astrocytes, characterized by hyper-responsiveness to glutamate stimulation and altered calcium flux. Interestingly, the effects of DHCR7 mutations appear to be the result of non-cell-autonomous influences from microglia, rather than being intrinsic to astrocytes themselves. This finding suggests that the interplay between astrocytes and microglia could be a contributing factor to the neurological symptoms observed in cholesterol biosynthesis disorders. Furthermore, these insights underscore a significant role for cholesterol metabolism within the astrocyte-microglia immune axis, potentially shedding light on mechanisms relevant to other neurological diseases.

The role of lipids, particularly the involvement of glyceronephosphate O-acyltransferase (GNPAT), is pivotal in understanding the onset of ASDs. GNPAT, which is crucial for ether phospholipid synthesis, is prominently expressed in astrocytes and microglia during post-natal development (Zhang et al., 2014, 2016; Bennett et al., 2016; Clarke et al., 2018; Düking et al., 2022). Its expression and function in these glial cells are significant due to their crucial roles in neurodevelopment and neural signaling. For instance, compromised GNPAT function in immature astrocytes could disrupt the lipid composition of the developing brain, potentially affecting neuronal development and signaling. Similarly, alterations in GNPAT function in immature microglia might impact their responsiveness to neuronal signals or their involvement in neuroinflammatory processes, both of which are implicated in the development of ASDs. Studies involving GNPAT knockout mice, which lack the ability to biosynthesize essential ether lipids including plasmalogens, have highlighted the enzyme’s critical role in manifesting symptoms typical of ASDs, such as impaired social interaction, repetitive behavior, and hyperactivity (Dorninger et al., 2019a,b). These observed behaviors in knockout mice are reflective of ASD-like symptoms, suggesting that plasmalogens – and by extension, GNPAT’s role in their synthesis – may be integral to the development of ASDs.

Mitochondrial dysfunctions are increasingly recognized as crucial contributors to the onset of inflammatory processes (Marchi et al., 2023). Mitochondria, beyond being the energy powerhouses of cells, play a pivotal role in regulating immune responses. When their function is compromised, it can lead to an accumulation of ROS and the release of mitochondrial DNA (mtDNA) into the cytoplasm. Both ROS and mtDNA act as potent triggers for inflammatory signaling pathways. Elevated ROS levels, for instance, can activate the NF-κB pathway, a central regulator of inflammation, resulting in the production of pro-inflammatory cytokines. Moreover, mitochondrial dysfunctions can disrupt cellular energy balance, affecting metabolic pathways such as glycolysis and the TCA cycle, and thereby exacerbating inflammatory responses. A shift toward glycolysis, often observed in immune cells during inflammation, is linked to mitochondrial impairments. This metabolic reprogramming, known as the Warburg effect, involves increased glucose uptake and lactate production, a phenomenon commonly seen in activated immune cells like macrophages and microglia. The altered metabolic state not only meets the energy demands of these cells but also contributes to the production of inflammatory mediators. Furthermore, there is a clear interrelationship between inflammation and glycolysis. Inflammatory responses necessitate the coordination of multiple players, including innate immune cells such as neutrophils and macrophages, as well as brain cell types like microglia. When activated, microglial cells undergo metabolic reprogramming, initially using glycolysis as an energy source during peak inflammation and later relying on OXPHOS metabolism during the resolution phase to adopt a pro-resolving phenotype (Gimeno-Bayón et al., 2014; Wang et al., 2019; Bernier et al., 2020). This indicates that different metabolic routes determine the fate of microglial cells and influence inflammatory responses.

Significantly, many studies have found that early-life inflammation is a risk factor for various neurodevelopmental disorders, including ASDs (Jiang et al., 2018). Animal models of maternal immune activation have provided insights into the cellular mechanisms of metabolic dysregulation (Estes and McAllister, 2016), highlighting the importance of prenatal inflammatory insults. However, recent research suggests that adverse experiences in early childhood, including inflammation, can also affect the risk of developing neurodevelopmental disorders. A recent study analyzed single-cell transcriptomic profiles of postmortem cerebella from children aged 1–5 years, both with and without inflammation (Ament et al., 2023). The findings revealed an increase in putative proinflammatory microglial cells expressing the IL1B and CD83 + genes in children experiencing inflammation. This suggests that brain tissue inflammation may be associated with a shift toward microglial subpopulations that express classical markers indicative of reactive proinflammatory states. Moreover, these inflammatory states were found to lead to a premature down-regulation of developmental gene expression programs. This included a significant decrease in the expression of genes previously implicated, through loss-of-function mutations, in increasing the risk for neurodevelopmental disorders. The decreased expression of these critical genes underscores the profound impact of early inflammatory states, providing a deeper understanding of how inflammation might predispose individuals to neurodevelopmental disorders. These insights emphasize the importance of targeting inflammatory pathways as a potential therapeutic strategy to mitigate the risk of such disorders. Other studies have identified mechanisms by which glycolysis influences pro-inflammatory gene transcription in microglia (Bernier et al., 2020). During inflammatory microglial activation, there are changes in substrate transport into cells, enzymatic regulation, and the transcription of metabolic genes. For example, increased glucose uptake by activated microglia may result from the up-regulation of GLUT1 (Bennett et al., 2016; Wang et al., 2019), HK1/2 (Bennett et al., 2016; Li et al., 2018), or PFKFB3, a key driver of aerobic glycolysis (Bennett et al., 2016; Nair et al., 2019). As PFKFB3 regulates glycolytic activity, transcriptional control of glycolytic machinery expression may depend on mTOR, a master regulator of metabolism implicated in both syndromic and idiopathic ASDs (Auerbach et al., 2011; Yecies and Manning, 2011; Saxton and Sabatini, 2017; Winden et al., 2018; Gazestani et al., 2019; Rosina et al., 2019). Activation of mTOR correlates with microglial reprogramming upon lipopolysaccharide (LPS) treatment, and its inhibition can block the LPS-induced increase in glycolysis (Hu et al., 2020). Additionally, LPS treatment increases the expression of hypoxia-inducible factor-1α (HIF-1α), a key gene coordinating the Warburg effect (York et al., 2021), inducing various glucose transporters and glycolytic enzymes such as aldolase A, suggesting that HIF-1α may regulate metabolic control during inflammatory conditions (Coleman and Blass, 1985).

## 5 Conclusions

The intricate interplay between astrocytes, microglia, and metabolic dysregulation in the context of ASDs reveals a complex landscape of potential contributors to the etiology of these neurodevelopmental conditions. The evidence presented underscores the importance of astrocytes and microglia, in the manifestation of ASDs-related deficits. The observed dysregulation in metabolic pathways, ranging from mitochondrial dysfunction to alterations in lipid metabolism and glycolytic shifts, adds a layer of complexity to our understanding of the biological mechanisms underlying ASDs. Future research endeavors should focus on unraveling the specific molecular pathways that link glial cell dysfunction and metabolic irregularities to ASDs pathophysiology. Targeting these pathways could provide novel therapeutic avenues for ASDs interventions. Additionally, investigating the impact of environmental factors, such as nutrition and inflammation, on glial cell function and metabolism during critical developmental periods may offer insights into preventive strategies. In the realm of future perspectives, leveraging evolving model systems, particularly those derived from human-induced pluripotent stem cells (hiPSC), holds tremendous promise in advancing our comprehension of the intricate relationship between astrocytes, microglia (Bezzi, 2022; Cordella et al., 2022; D’Antoni et al., 2023), and metabolic pathways in the context of ASDs. The development and integration of hiPSC-derived microglia and astrocytes into experimental frameworks offers an unprecedented opportunity to investigate the dynamic interplay of these glial cells in a human-specific context. This approach enables the recreation of patient-specific cellular environments, allowing researchers to dissect the specific contributions of astrocytes and microglia to metabolic dysregulation in ASDs. Furthermore, the adoption of cutting-edge methodologies, including single-cell analyses, spatial transcriptomics, and multi-omics approaches, can provide a more granular and comprehensive understanding of the cellular and molecular mechanisms orchestrating ASD pathology. Advanced imaging techniques, such as high-resolution microscopy and live-cell imaging, contribute to capturing the dynamic nature of glial cell interactions and metabolic processes. Integrating these sophisticated tools not only refines our current understanding but also opens new avenues for the identification of potential therapeutic targets and personalized treatment strategies tailored to the specific metabolic signatures associated with ASDs subtypes.

## Author contributions

IC: Writing – review & editing. CC: Writing – review & editing. GD: Writing – review & editing. CL: Writing – review & editing. PB: Conceptualization, Funding acquisition, Writing – original draft, Writing – review & editing.
